# P-wave Cooper pair splitting

**DOI:** 10.3762/bjnano.3.56

**Published:** 2012-07-06

**Authors:** Henning Soller, Andreas Komnik

**Affiliations:** 1 Institut für Theoretische Physik, Ruprecht-Karls-Universität Heidelberg, Philosophenweg 19, D-69120 Heidelberg, Germany

**Keywords:** Cooper pair splitting, entanglement, Hamiltonian approach, spin-active scattering, superconductivity

## Abstract

**Background:** Splitting of Cooper pairs has recently been realized experimentally for s-wave Cooper pairs. A split Cooper pair represents an entangled two-electron pair state, which has possible application in on-chip quantum computation. Likewise the spin-activity of interfaces in nanoscale tunnel junctions has been investigated theoretically and experimentally in recent years. However, the possible implications of spin-active interfaces in Cooper pair splitters so far have not been investigated.

**Results:** We analyze the current and the cross correlation of currents in a superconductor–ferromagnet beam splitter, including spin-active scattering. Using the Hamiltonian formalism, we calculate the cumulant-generating function of charge transfer. As a first step, we discuss characteristics of the conductance for crossed Andreev reflection in superconductor–ferromagnet beam splitters with s-wave and p-wave superconductors and no spin-active scattering. In a second step, we consider spin-active scattering and show how to realize p-wave splitting using only an s-wave superconductor, through the process of spin-flipped crossed Andreev reflection. We present results for the conductance and cross correlations.

**Conclusion:** Spin-activity of interfaces in Cooper pair splitters allows for new features in ordinary s-wave Cooper pair splitters, that can otherwise only be realized by using p-wave superconductors. In particular, it provides access to Bell states that are different from the typical spin singlet state.

## Introduction

Solid-state entanglers represent electronic analogues to the optical setups used for Bell inequality tests. Most setups propose a superconductor as a source of spin-entangled s-wave Cooper pairs [1–3]. These are transferred to spatially separated leads by the process of crossed Andreev reflection (CAR) [4–5]. Different setups have been considered in order to achieve CAR without being dominated by elastic cotunneling or ordinary Andreev reflection (AR) processes [6–9]. S-wave Cooper pairs are entangled in energy and spin space. Therefore, one may either filter the electrons of a Cooper pair in spin space (using ferromagnets [10–12] or Luttinger liquids [2]), or in energy space (using quantum dots [1,13], coupling to an electromagnetic mode [14], an appropriate voltage bias [15–18] or ac-bias [19]). Filtering using quantum dots with large onsite interaction has been successfully realized in experiments [20–22] and a nonlocal resistance has been measured as a characteristic of Cooper pair splitting. Moreover, a positive cross correlation measured in a superconductor and normal-metal three-terminal device gave compelling evidence for CAR [23]. However, s-wave Cooper pairs only give access to one of the Bell states, namely 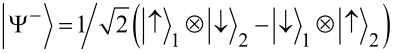, where the subscripts 1 and 2 refer to two normal leads. P-wave Cooper pairs give access to the other Bell states, especially those such as 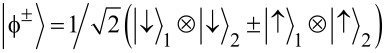, involving fully spin-polarized combinations of the two electrons. Thus, a p-wave Cooper pair splitter represents the essential counterpart to s-wave Cooper pair splitters as on-chip sources of spin-entangled Einstein–Podolsky–Rosen (EPR) electron pairs [24–26].

**Figure 1:**
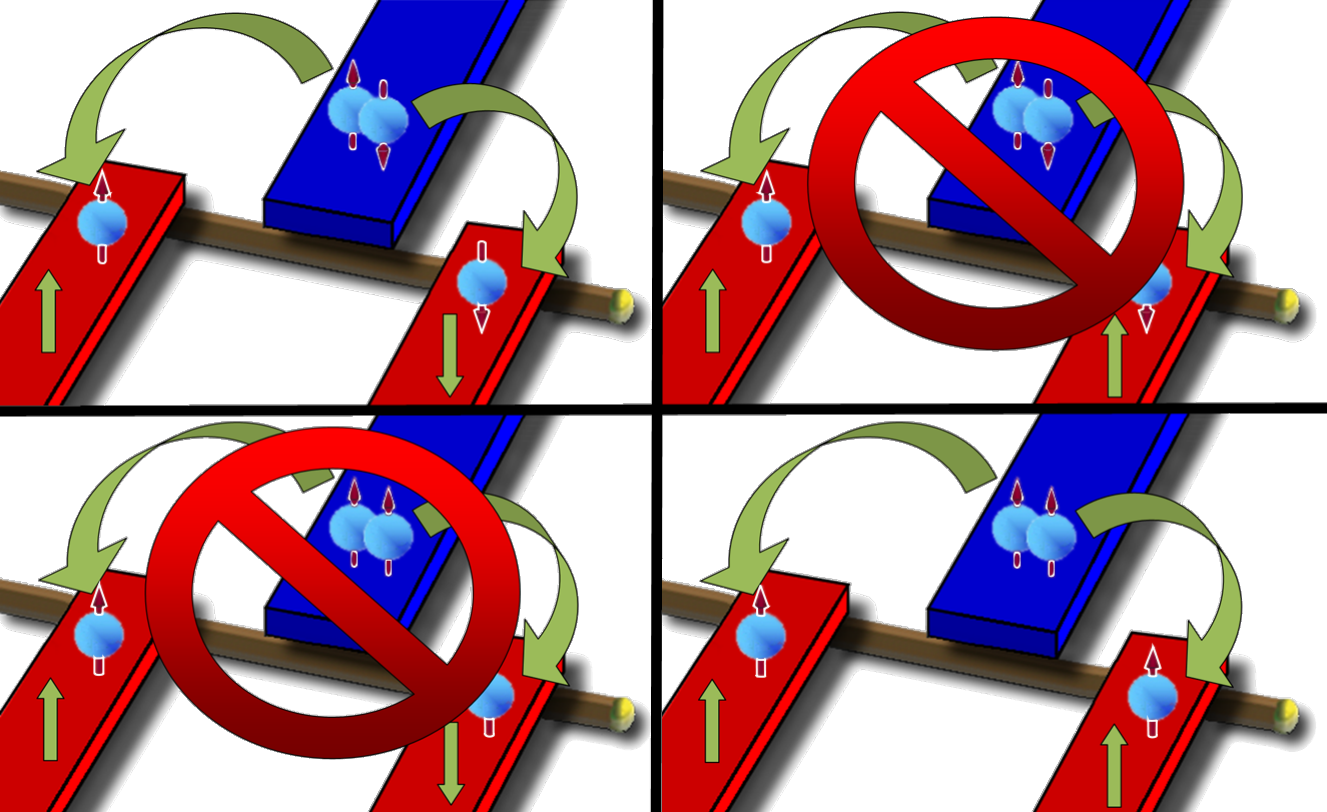
Summary of the possible charge-transfer processes in a superconductor–ferromagnet beam splitter. The superconductor (blue) and the two ferromagnets (red, assumed to be fully polarized) are coupled via a quantum dot, which is realized by an InAs nanowire (brown). The polarization is indicated by green arrows. In the upper part, the situation for s-wave superconductors is shown. The Cooper pair may split if the two ferromagnets are antiparallelly polarized. In the lower part the reversed situation for spin-polarized p-wave superconductors is depicted. The Cooper pair may now split only if the two ferromagnets are equally polarized.

In this paper we approach the problem of p-wave Cooper pair splitting in two steps. First, we show that p-wave splitting may easily be identified in a hybrid junction between a superconductor and two ferromagmets (setup shown later in Figure 1). However, p-wave superconductors that can be easily handled in quantum transport experiments are presently not available. Therefore, in a second step we show how p-wave splitting can be realized without using a p-wave superconductor. Indeed, the previous works on the charge transfer statistics of superconductor ferromagnet beamsplitters [10,27–28] neglected the recently predicted [29–31] and observed [32–33] effects of the interface on charge transfer. By including a better description of the interface one can describe the shared triplet pairs formation between the ferromagnets. We calculate the full counting statistics (FCS), which allows us to identify the corresponding charge-transfer process and to calculate the cross correlation as an experimentally observable quantity [23].

## Results and Discussion

### Superconductor–ferromagnet beam splitters

Splitting of spin-polarized p-wave Cooper pairs can easily be identified in the conductance. From the result in [27] we find the generalization of Beenakker’s formula [34] for the zero-bias conductance of a beam splitter realized by a resonant level between a superconductor and two ferromagnets:

[1]
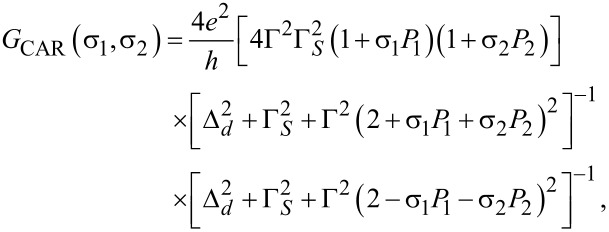


where Γ is the tunnel rate through the two barriers between the quantum dot and the ferromagnets (both are assumed to be equal) and Γ*_S_* is the tunnel rate between the superconductor and the quantum dot. Δ*_d_* refers to the energy of the resonant level. *P*_1_ and *P*_2_ are the (parallel) polarizations of the ferromagnets and σ_1_ and σ_2_ are the spins of the electrons in a Cooper pair.

In usual s-wave superconductivity the spin directions obey σ_1_ = −σ_2_, and we may maximize CAR by choosing *P*_1_ = 1 = −*P*_2_ (or vice versa) [10]. However, if we choose the polarizations of the ferromagnets to be equal (i.e., *P*_1_ = 1 = *P*_2_) *G*_CAR_ in Equation 1 becomes zero (Figure 1). The situation is reversed if we introduce a spin-polarized p-wave superconductor such that σ_1_ = σ_2_. Now splitting is maximized if *P*_1_ = *P*_2_. Of course now for antiparallel polarization the current is blocked (Figure 1). Therefore p-wave splitting is easily identified in the crossed conductance. Maximal polarization is not easy to realize in experiment [35] but we use this assumption to illustrate the argument. In spite of the apparent simplicity of this consideration a serious problem remains: P-wave superconductors are very rare and materials such as Sr_2_RuO_4_ are hard to handle [36].

However, recent theoretical and experimental progress showed that the treatment of superconductor–ferromagnet interfaces requires special care with respect to the exact form of the interface and the associated spin-active nature of tunneling [37]. In the rest of this work we want to show how a spin-active interface can be used in exactly the same way as a p-wave superconductor and therefore allows the generation of the other Bell states. In order to accomplish this goal we will show how the transport characteristics of p-wave splitting can be imitated by spin-active scattering.

### Cumulant-generating function with spin-active scattering

To identify the separate charge-transfer processes and to evaluate the conductances and cross correlations, we calculate the FCS of charge transfer [38–39] using the generalized Keldysh technique [40–42]. The aim is to calculate the cumulant-generating function (CGF) ln χ(λ) of the probability distribution *P*(*q*) to transfer *q* units of charge during a (long) measurement time τ: χ(λ) = Σ*_q_*e*^i^*^λ^*^q^P*(*q*). Partial derivatives of this function provide direct access to the cumulants (irreducible moments) of *P*(*q*).

We model the superconductor–ferromagnet beam splitter as two ferromagnets *F*1 and *F*2 that are tunnel-coupled to a resonant level, which is the simplest model of a quantum dot. The superconductor is also coupled to the resonant level via *H_T_*_1_ and *H_T_*_2_, which takes into account the interface effects. The result is the Hamiltonian

[2]
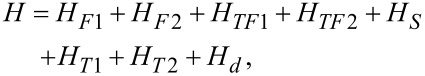


where

[3]
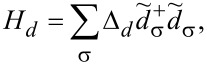


represents the resonant level. Throughout the rest of this exposition we use units in which *e* = 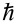 = *k*_B_ = 1 and restrict ourselves to a quantum dot on resonance Δ*_d_* = 0.

Ferromagnetic electrodes are described in the language of electron field operators Ψ*_k_*_,α,σ_, where α = *F*1, *F*2 using the Stoner model with an exchange energy *h_ex_* as in [43]

[4]
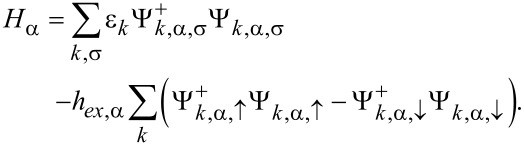


Consequently they can be described as fermionic continua with a spin-dependent density of states (DOS) ρ_0,α,σ_ = ρ_0,α_(1 + σ*P*_α_), where *P*_α_ is the polarization. The superconductor is described by using the ordinary s-wave BCS Hamiltonian

[5]
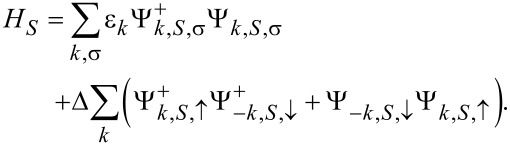


We express the energies of the dot and the reservoirs relative to the superconductor chemical potential [44] such that μ*_S_* = 0 and *V*_α_ = μ*_S_* − μ_α_ = −μ_α_ is the chemical potential of the ferromagnets. The quasiparticle density of states in the superconductor has the form: 
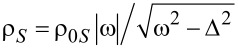
, where ρ_0_*_S_* is constant in the wide-band limit. Tunneling between the superconductor and the quantum dot is given by [45]

[6]
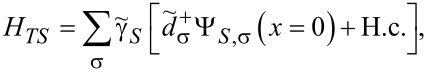


where 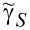 is the corresponding tunneling amplitude between the dot and the superconductor. The tunneling is assumed to be local and to occur at *x* = 0 in the coordinate system of the respective electrode. Ψ*_S_*_,σ_(*x*) refers to the electron field operator of the superconductor introduced in Equation 5 in position space.

Finally, we need a Hamiltonian approach [46] for spin-active scattering. There are manifold effects, such as spin–orbit coupling, magnetic anisotropy or spin relaxation, that give rise to spin-activity of interfaces [29]. Previous studies of point contacts used a scattering states description [29–31,47] in order to introduce a spin-active scattering angle as a phenomenological parameter to characterize the interface or a wave-function-matching technique [48–49]. We adopt the simplest possible approach and follow [50–52] by introducing two tunneling Hamiltonians at each of the interfaces between the quantum dot and a ferromagnet

[7]
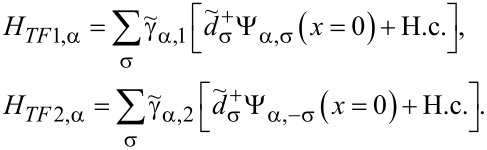


*H_TF_*_1,α_ describes normal spin-conserving tunneling whereas *H_TF_*_2,α_ refers to the spin-flip processes [Ψ_α,−σ_(*x* = 0) represents an electron in the ferromagnet with opposite spin]. If we take spin-active scattering into account in this way we have five different tunnel couplings. In order to reduce the number of parameters and for clarification of the discussion of spin-active scattering effects we want to limit ourselves to a special constellation of parameters as far as spin-active scattering is concerned, namely γ*_F_*_1,1_ = γ*_F_*_2,1_, γ*_F_*_1,2_ = γ*_F_*_2,2_. In this case we define operators

[8]
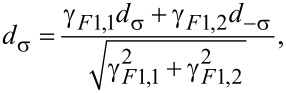


which allow us to rewrite the tunneling Hamiltonians in Equation 6 and Equation 7 as

[9]
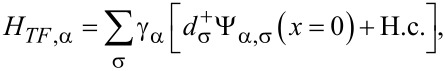


[10]
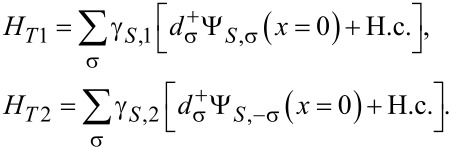


This minimal model still reveals all of the transport properties that we wish to discuss here. In order to access the CGF, we introduce the counting fields for the leads attached to the quantum dot as time-dependent fictitious parameters, that is, counting fields in front of the creation and annihilation operators of the respective electrodes

[11]
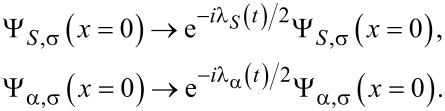


λ_(_*_S_*_,1,2)_(*t*) takes the value +(−)λ_(_*_S_*_,1,2)_ on the forward/backward branch of the Keldysh contour 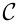. The counting fields are only switched on during the measurement time τ. In the limit of long measurement times τ, the CGF can be calculated analytically by using a generalized Green’s function formalism that has previously been used to calculate the FCS of other quantum impurity systems [53–55]. Following [56] the CGF is given by the expectation value

[12]
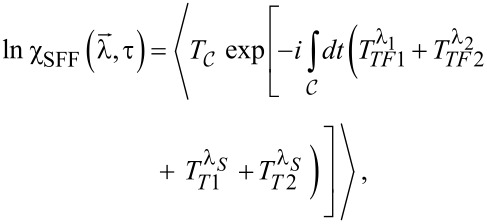


where 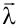 = (λ_1_, λ_2_, λ*_S_*) and 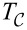 denotes the contour time ordering operator. 
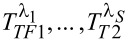
 are abbreviations for the tunneling operators introduced in Equation 9 and Equation 10 in combination with the substitutions defined in Equation 11.

For the FCS calculation in the limit of large measurement times (as assumed here) we follow [40] and write the above expression using an adiabatic potential [57], which, in the limit of infinitely long measuring time, is time-independent


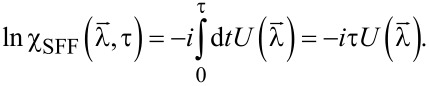


In turn the adiabatic potential is related to the counting field derivatives of the *T*^λ^’s introduced in Equation 12


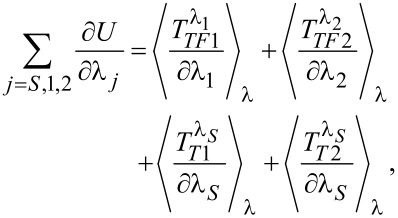


where 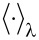 is defined as 
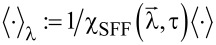
 with 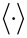 being the ordinary expectation value with respect to the systems Hamiltonian in Equation 2, with the tunneling Hamiltonians rephrased by using the substitution defined in Equation 11. The derivatives of the *T*^λ^’s take the form of mixed and 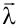-dependent Green’s functions, e.g.,


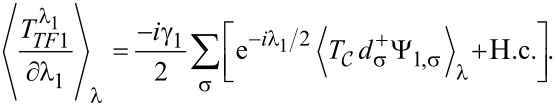


These mixed Green’s functions have to account for all orders of the tunneling coupling. By expanding them to first order in the relevant tunnel coupling they can all be rewritten as a product of a bare-lead Green’s function (subscript 0) and an exact in-tunneling-dot Green’s function. E.g., for the derivative considered above


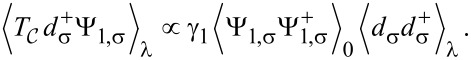


Therefore the remaining task is an exact calculation of the dot Green’s function. Since the system’s Hamiltonian in Equation 2 is quadratic the Dyson equation can be solved exactly. However, as also noted in [58], coupling of the dot to the superconductor automatically leads to the appearance of anomalous dot Green’s functions of the type 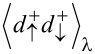 to second order in the tunnel coupling to the superconductor. Additionally, the spin-flipping tunnel contribution in Equation 10 gives rise to correlation functions of the type 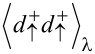 [59]. Consequently the full dot Green’s function for the spin species ↑ becomes a four-component vector of correlation functions 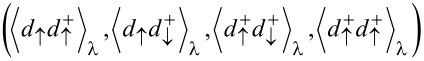. Since the counting fields take different signs on the backward/forward branch of the Keldysh contour each correlation function is a 2 × 2 Keldysh matrix again. The bare-dot and lead Green’s functions are, e.g., given in [60]. Since the solution of the Dyson equation allows all orders to be summed up in the tunneling couplings, the result is exact and also valid at finite temperature, as discussed also in [46].

However, the result for the average in Equation 12 is quite long. We want to restrict ourselves to the study of the relevant aspects of the CGF in view of the possibility of p-wave splitting only. Comparing our method to previous treatments of superconductor hybrids, we should emphasize that also in the quasiclassical Green’s function formalism [61] calculations of the current exact-in-tunneling have been performed [62–63]. However, in that case it was assumed that there were two tunneling contacts to normal leads, with large spatial separation. In our case the three leads are coupled via a quantum dot, which corresponds to the opposite limit and involves an energy-dependent transmission. Therefore the setup considered here is more closely related to the studies in [58] and [64] in which, however, a disordered quantum dot and no spin-active scattering was assumed, whereas here we treat the ballistic case with spin-active scattering. Disorder for the case of tunnel contacts was considered by using the Keldysh–Usadel formalism in [65], [66] and [67]. Additionally we want to point out that the description of spin-active scattering used here and in [63] and [29] is different: In the previous works the spin-mixing angle is introduced as a phenomenological parameter, whereas we use a second tunneling transparency to account for spin flips. For a comparison of both descriptions of tunnel contacts we refer the reader to [68].

### Spin-flipped crossed Andreev reflection

In simple superconductor-ferromagnet tunneling junctions the presence of spin-active scattering gives rise to a new type of AR. In ordinary AR an electron is retroreflected as a hole with opposite spin since Cooper pairs represent spin singlets. However, due to the spin-active nature of tunneling in the setup considered here, the hole or the electron spin can be flipped. This spin-flipped Andreev reflection (SAR) induces triplet correlations in the ferromagnet [29]. To see whether similar effects occur in our setup we calculate the zero-bias conductance analogously to Equation 1 for the case of *P*_1_ = *P*_2_ = 1, meaning maximal parallel polarization


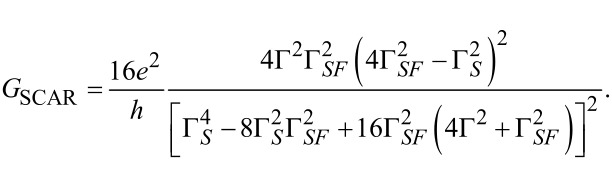



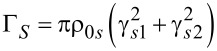
 and 
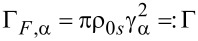
 again refer to the tunnel rates for ordinary single electron tunneling. Γ*_SF_* = πρ_0_*_s_*γ*_s_*_1_γ*_s_*_2_ describes the additional spin-flip tunneling rate at the interface. Obviously, spin-active scattering has lifted the current blocking indicated in Figure 1 for a s-wave superconductor connected to two maximally parallelly polarized ferromagnets. Therefore this finite conductance is similar to the one obtained for a p-wave superconductor junction in Equation 1. This indicates that this conductance for voltages below the gap is indeed associated with a spin-flipped crossed Andreev reflection (SCAR) in which a triplet pair is transferred to the ferromagnets.

Of course one may also obtain this information from the CGF itself. However, the expression is complicated and the probability distribution of charge transfer is also not easy to access in an experiment [69–70]. Therefore we follow [3] and use the cross correlation as an indication to probe whether the two charges of a Cooper pair transferred in a SCAR process are indeed transferred to the ferromagnets at the same time. The presence of nonzero *G*_SCAR_ at voltages below the gap and *T* = 0 in combination with a positive cross correlation can only be explained by a simultaneous transfer of a triplet pair to the ferromagnets, which implies that we indeed observe p-wave splitting. The cross correlation can be calculated as a mixed second derivative of the CGF

[13]
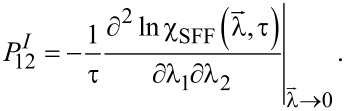


**Figure 2:**
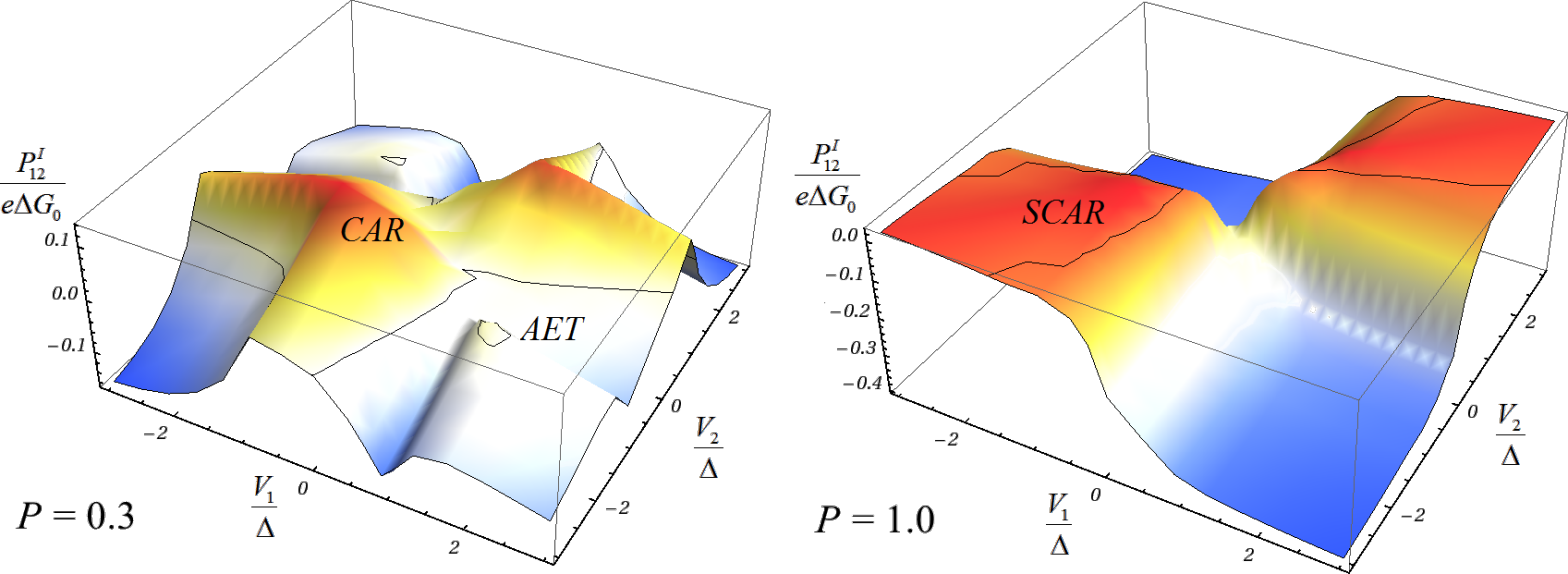
Cross correlation of currents for different parameters: The cross correlation 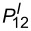 according to Equation 13 is calculated for two different sets of parameters. The polarization of the ferromagnets is assumed to be equal in both cases. The left graph shows the result for *P* = 0.3, Γ*_S_* = 4Δ, Γ*_SF_* = 0, Γ*_F_*_1_ = 0.4Δ, Γ*_F_*_2_ = 0.1Δ and *T* = 0.1Δ. The right graph is for *P* = 1, Γ*_S_* = 2Δ, Γ*_SF_* = Δ, Γ*_F_*_1_ = 0.05Δ = Γ*_F_*_2_ and *T* = 0.1Δ. A distortion of the superconducting DOS described by a Dynes parameter [72] of Γ*_D_* = 0.005Δ has been introduced in order to circumvent numerical artifacts of the diverging superconductor DOS. The cross correlations are positive in the deep-red regions.

In Figure 2 the result of Equation 13 is shown for two different configurations of the couplings and polarizations. For moderate polarization (*P* = 0.3) and no spin-active scattering, we find two cases in which positive cross correlation can be observed, in accordance with previous results [27,71]. First, for voltages close to the superconducting gap and *V*_1_ ≈ −*V*_2_, CAR is strongly suppressed and one expects single-electron transmission to be dominant. Nonetheless, the energy-dependent DOS of the superconductor leads to large transmission coefficients for double AR from one ferromagnet to the superconductor and further to the second ferromagnet. This process is known as Andreev reflection enhanced transmission (AET) [27]. The second case of positive cross correlation can be observed for one bias voltage being close to zero and a finite bias on the second electrode. In this case, CAR dominates over single-electron transmission and induces positive cross correlation [27].

This picture changes dramatically if we go over to the case of full polarization (*P* = 1) and finite spin-active scattering. Since AET relies on double AR, and consequently the spin symmetry of AR, it must disappear since SAR violates spin-symmetry and it would be the only possible charge-transfer process for a single lead for *P* = 1. However, for *V*_1_ ≈ *V*_2_ a positive cross correlation remains. This is exactly the position where we assume SCAR to be dominant since *V*_1_ ≈ *V*_2_ means that single-electron transfer between the ferromagnets does not occur. The effect is, of course, still observable for *P* < 1 but the polarization should be rather strong. Spin-active scattering in superconductor–ferromagnet hybrids is a general phenomenon [32] and full polarization was just assumed for clarity. Therefore, we believe that SCAR is a generic phenomenon that should also be present in superconductor–ferromagnet beam splitters with tunnel contacts [6,63] or chaotic cavities [58,64], since its origin does not lie in the precise form of the energy dependence of the transmission coefficients.

Experiments in the direction of the above-described proposal have already been realized [73]. Multiterminal hybrid systems with embedded quantum dots [74] also based on InAs nanowires [26,75] have already been realized experimentally. In such devices a new subgap structure has been observed, which can be explained by SAR [68]. This is of special importance, since in our consideration we did not include the effects of Coulomb interaction on the dot, and thus we should worry about a possible suppression of SCAR. The mean-field analysis of [68], however, revealed that SAR and therefore also SCAR should be observable also in the presence of strong Coulomb interaction. Apart from that, we can argue that also in interacting systems characteristic resonances, such as that of the resonant level considered here, are present and have a characteristic location and width associated with interactions. Therefore the general scenario should be robust. However, one should bear in mind that for more extended quantum dots or nanowires disorder could play an important role as mentioned in [65] and [66]. Another experiment realized a superconductor–ferromagnet–superconductor junction based on Al and Co electrodes with two closely spaced cobalt wires bridging two aluminum electrodes [76]. In this experiment the resistance in the case of antiparallel and parallel magnetization of the two wires was measured, and for low temperatures it was found that the antiparallel arrangement may even have higher resistance than the parallel one, giving reliable evidence for spin-active scattering being present in the device. Concerning a possible experimental realization using quantum dots one should consider that the interaction should be small enough and the polarization large enough so as not to completely suppress SCAR, and the coupling to the leads should be of the order of the superconductor gap Γ, Γ*_S_*, Γ*_SF_* ≈ Δ. Nowadays, this coupling is generally obtained in experiments using InAs nanowires or carbon nanotubes as quantum dots [77].

## Conclusion

To conclude, we considered superconductor–ferromagnet beam splitters without a specific consideration of interface properties. We found that splitting of spin-polarized p-wave Cooper pairs can easily be identified in the conductance. However, p-wave superconductors that are usable in experiments are not available, thus we proposed a scheme to mimic their behavior by taking into account the spin activity of superconductor–ferromagnet interfaces. The newly identified SCAR process allows one to obtain split p-wave Cooper pairs, which gives access to the Bell states other than 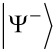.
